# Enhanced sweet taste perception in obesity: Joint analysis of gustatory data from multiple studies

**DOI:** 10.3389/fnut.2022.1028261

**Published:** 2022-12-20

**Authors:** Gabriela Ribeiro, Sandra Torres, Ana B. Fernandes, Marta Camacho, Teresa L. Branco, Sandra S. Martins, Armando Raimundo, Albino J. Oliveira-Maia, Food Reward in Bariatric Surgery Portuguese Study Group

**Affiliations:** ^1^Champalimaud Research and Clinical Centre, Champalimaud Foundation, Lisbon, Portugal; ^2^Lisbon Academic Medical Centre PhD Program, Faculdade de Medicina da Universidade de Lisboa, Lisbon, Portugal; ^3^Faculdade de Psicologia e de Ciências da Educação, Universidade do Porto, Porto, Portugal; ^4^Centro de Psicologia da Universidade do Porto, Porto, Portugal; ^5^NOVA Medical School, Faculdade de Ciências Médicas, NMS, FCM, Universidade NOVA de Lisboa, Lisbon, Portugal; ^6^Exercise and Health Laboratory, CIPER, Faculdade de Medicina da Universidade de Lisboa, Cruz Quebrada, Portugal; ^7^Universidade Europeia, Lisbon, Portugal; ^8^Instituto de Saúde Ambiental (ISAMB), Faculdade de Medicina da Universidade de Lisboa, Lisbon, Portugal; ^9^Departamento de Desporto e Saúde, Escola de Saúde e Desenvolvimento Humano, Universidade de Évora, Évora, Portugal; ^10^Comprehensive Health Research Centre (CHRC), Universidade de Évora, Évora, Portugal

**Keywords:** obesity, sweet taste, taste perception, gustation, food reward, hedonic hunger, reward-related feeding behavior

## Abstract

**Introduction:**

While sweet taste perception is a potential determinant of feeding behavior in obesity, the supporting evidence is inconsistent and is typically associated with methodological limitations. Notably, possible associations between sweet taste perception and measures of food reward remain undetermined.

**Materials and methods:**

We conducted a cross-sectional analysis comparing 246 individuals with severe obesity and 174 healthy volunteers using a validated method for taste perception assessment. We included gustatory variables, namely intensity and pleasantness ratings of sour, salt, sweet, and bitter tastants, and taste thresholds assessed by electrogustometry. Reward-related feeding behavior, including hedonic hunger, food addiction, feeding behavior traits, and acceptance of foods and alcohol, was evaluated using self-rated scales for comparison with gustatory measures.

**Result:**

In logistic regressions adjusted for age, gender, educational level, and research center, we found that a greater likelihood of belonging to the obesity group was associated with higher sweet intensity ratings (OR = 1.4, *P* = 0.01), hedonic hunger, food addiction symptoms, restrained and emotional eating (1.7 < OR ≤ 4.6, all *P* ≤ 0.001), and lower alcohol acceptance (OR = 0.6, *P* = 0.0002). Using principal component analysis, we found that while hedonic hunger, food addiction, and emotional eating were strongly interrelated, they were not associated with sweet intensity perception that, in turn, had a closer relationship with alcohol acceptance and restrained eating.

**Conclusion:**

We found that individuals with obesity report higher sweet taste intensity ratings than healthy controls. Furthermore, while psychological measures of reward-related feeding behavior assess a common construct, sweet intensity perception may represent a different obesity-related dimension.

## Introduction

Obesity is a global and complex health concern (1, 2), with increasing prevalence (3, 4) and severe socio-economic repercussions (1, 2, 5, 6). The high availability of foods rich in sugar or fat is implicated in the obesity epidemic (1, 7, 8). Sugar, *via* its pre- and post-ingestive value (9–12), acts in brain reward circuitries, inducing food preferences (10, 12) and food-seeking behaviors (11).

Ingestive behavior is multifactorial, aggregating several appetitive processes such as incentive motivation, “wanting,” “liking,” and reinforcement learning, with underlying neurobiology that is not fully overlapping (13, 14). Additionally, terms like “liking” are frequently used interchangeably with “preference,” although they are tested using distinct behavioral paradigms and reflect fairly distinct concepts. “Liking” is typically a self-reported perceptual experience obtained from ratings of how pleasant or unpleasant a stimulus is, according to a standard scale (13–15). For reference, perception occurs when sensory signals (e.g., sweet stimulus) are interpreted and integrated into the central nervous system to produce a conscious experience, such as a liking or pleasantness perception (16). On the other side, preference is commonly determined by a choice between two or more alternatives, classically with a procedure to track choice for, and/or consumption of, the several stimuli (i.e., tastant, food, or beverage) (13–15). Preference and liking are not necessarily overlapping constructs. Indeed, others have argued that, when exposed to a series of beverages with increasing sweetness, the individuals that prefer the sweetest beverage do not necessarily perceive sweetness as more pleasurable, and participants preferring less sweet beverages may actually have higher liking ratings across the several options. Furthermore, variability in preference for different levels of sweetness does not result necessarily from intensity coding of taste, which is a factor of neural responses to tastants, that increase with stimulus concentration and correlate with perceived taste intensity (17). Thus, behavioral preferences for sensations elicited by greater sweetness, which is a proxy for higher energy densities, do not necessarily reflect increased hedonic (i.e., conscious, and subjective liking/pleasantness) responses (13, 14).

The literature about taste perception in human obesity has been predominantly focused on liking for appetitive tastants. While the view that individuals with obesity like sweet taste more than normal weight individuals is still prevailing (14), previous work on sweet taste perception and obesity (13, 14, 18) has led to inconsistent findings (19). For example, some studies that used direct measures of taste, including appetitive (sweet) and non-appetitive (salt, sour, and bitter) tastants, to compare individuals with obesity with non-obese controls, reported absent associations between several taste perception parameters and obesity (20–24). In contrast, others found either negative (25–27) or positive (28–30) associations, including for sweet taste (28, 30, 31). Furthermore, there was significant methodological heterogeneity across studies (19, 32), including for stimuli type and gustatory outcomes (19). For example, sweet taste outcomes varied from intensity and pleasantness ratings using several distinct scales (e.g., 9-point scale, visual analogue scales, and general labelled magnitude scales), to detection and recognition thresholds, as well as the “preferred concentration” (19). For sweet pleasantness and intensity ratings, early studies did not find consistent obesity-dependent differences for intensity ratings, with one study suggesting that individuals with obesity rated higher concentrations of sweet as more pleasant (28), and another study suggesting that individuals with obesity rated higher concentrations of sucrose as less pleasant (25). In another study, adolescents with obesity rated sweet and salty tastants as more intense, and also rated the lowest NaCl solution as less pleasant, with no pleasantness differences for sucrose (29). In a more recent study, individuals with obesity rated the lower concentrations of sucrose, NaCl and citric acid as more intense, and one of the higher concentrations of sucrose as more pleasant, relative to normal weight participants (31). Several other studies did not find differences in neither pleasantness nor intensity ratings (20, 21, 24, 30).

Across these studies there are other limitations (19), such as small sample sizes, general lack of a control group in longitudinal studies (19), and a general absence of feeding behavior-related correlates. These factors haltered an adequate estimation of taste perception contribution toward obesity. We recently demonstrated that sweet intensity perception predicted weight loss following bariatric surgery in over 200 patients (33). Although surgery did not induce a generalized change in taste perception, baseline sweet intensity ratings were positive predictors of weight loss 18 months after surgery. Also, patients that decreased intensity ratings for sweet stimuli lost more weight (33). Steele and colleagues also showed that taste-related reward processing predicted weight loss at 6 months for gastric bypass but not sleeve gastrectomy patients (34). However, taste-related reward processing induced by gastric bypass changes may be temporary and dependent upon post-operative eating behaviors (35). It is thus necessary to better characterize reward-related feeding behavior in obesity, including gustation and obesogenic behaviors. Here we hypothesized that altered sweet taste perception (i.e., intensity and pleasantness) is associated with obesity. To address this hypothesis, while avoiding the previous limitations, we included a large group of individuals with obesity and a group of healthy volunteers as controls. In exploratory analyses, we tested if sweet taste perception was associated with psychometric measures of reward-related feeding behavior.

## Materials and methods

We conducted a cross-sectional analysis to compare a group of individuals with obesity (obesity group), including pre-bariatric patients (33) and participants of “*Peso Pesado”* (the equivalent to “*The Biggest Loser”* in Portugal), with healthy volunteers (healthy group). All groups had data regarding height, weight, and gustatory variables, including acuity in taste identification, intensity, and pleasantness ratings given to basic tastants (i.e., sour, salt, sucrose, and bitter) and taste detection thresholds. In addition, all groups except the “*The Biggest Loser”* participants had self-rated psychometric scales of reward-related feeding behavior.

### Study design and participants

The recruitment of bariatric surgery candidates took place consecutively at three tertiary care outpatient centers specialized in the surgical treatment of obesity in Portugal (Hospital do Espírito Santo EPE, Évora; Hospital de São Bernardo, EPE, Setúbal; Centro Hospitalar Universitário de São João EPE, Porto). The cohort included patients approved for bariatric surgery following the Portuguese National Health Service criteria. We collected data from this cohort for longitudinal purposes (Trial registration number: ISRCTN59323751), as previously described (22). “*The Biggest Loser”* subgroup was recruited from two seasons in Portugal (2011 and 2012), both assessed before and after the weight-loss intervention. In addition, healthy individuals were recruited from the community by the two research centers involved in the study, namely Champalimaud Research and Clinical Centre (Lisbon, Portugal) and Faculdade de Psicologia e Ciências Educação da Universidade do Porto (Porto, Portugal). All groups had equivalent exclusion criteria, including active acute respiratory infection, active neurological or psychiatric disease, active gastrointestinal, hepatic or pancreatic disease, illicit substance use or alcohol abuse, illiteracy or inability to understand the study’s instructions, prior major gastrointestinal surgery, intra-gastric balloon in the previous 12 months, history of food allergies and pregnancy or breastfeeding. In healthy controls, diabetes and obesity (defined as body mass index–BMI ≥ 30 Kg/m^2^) were additional exclusion criteria. The study followed the principles of the Declaration of Helsinki and was approved by local Ethics Committees at each participating institution. Written informed consent was obtained from all participants.

### Measures

The study protocol included a health questionnaire, followed by measurements of weight and height obtained with digital scales and stadiometers (Seca, Hamburg, Germany). The BMI was calculated as weight in kilograms divided by height in meters squared.

The taste strips test used in this study follows a validated and published protocol [Landis et al. (36)]. Since the taste strips were produced in house and considering the high methodological variability in gustatory measures, we validated our in-house taste strips test, determining temporal reliability and agreement with a commercially available version. The result of this methodological sub-study is described in Supplementary Information.

The gustatory test consisted of taste strips (i.e., filter paper) impregnated with a solution of one of the four basic tastants, namely citric acid (sour), sodium chloride (salt), sucrose (sweet), or quinine hydrochloride (bitter), or deionized water (details on the taste strips preparation are described in Supplementary Methods). The taste test follows a standardized protocol in which each tastant is presented in four increasing concentrations (36), in randomized order, except for quinine, which was always presented last. Following stimulation with each strip, subjects were asked to rate each tastant regarding intensity and pleasantness.

Intensity was rated using 100 mm vertical line general Labeled Magnitude Scale (gLMS) (37) ranging from 0 (labeled “without any sensation”) to 100 (labeled “the strongest sensation that I can imagine”) with five intermediate labeled levels (i.e., “barely detectable,” “weak,” “moderate,” “strong,” and “very strong”) (37). It was explained that this could refer to all kind of sensations, including pain. The pleasantness general Labeled Hedonic Scale (gLHS) (38) was a 200 mm vertical line scale, corresponding to a range of negative (−100, labeled “most unpleasant sensation that I can imagine”), neutral (0, labeled “neutral”) and positive (100, labeled “most pleasant sensation that I can imagine”) assessments, including eight other intermediate labels (i.e., four positive descriptors–“like slightly,” “like moderately,” “like very much,” “like extremely,” and four negative descriptors–“dislike slightly,” “dislike moderately,” “dislike very much,” “dislike extremely”) (38). Participants were instructed to rate the tastants in the context of the broadest possible range of pleasure and displeasure they experienced previously. Finally, participants were asked to identify the taste quality of that strip from a list of descriptors in a 5-alternative forced-choice test.

The primary outcomes of the gustatory protocol were mean intensity and pleasantness ratings given to the four concentrations of each tastant. Taste acuity was calculated as the mean number of correctly identified taste qualities in each trial (0 to 16). Finally, individual taste thresholds were assessed with electrogustometry (EGM) (39) using a commercially available electrogustometer (Rion TR-06; Rion Co. Ltd., Tokyo, Japan). Further details about methods were previously reported (19).

Reward-related feeding behavior was assessed using psychometric self-rated scales. We used the Power of Food Scale (PFS) (40, 41) and the Yale Food Addiction Scale (YFAS) (42), previously validated for the adult Portuguese population by our group (43–45). The PFS assesses hedonic hunger (i.e., the motivation to obtain food even in the absence of energy needs) and comprises three subscales that reflect increasing proximity to food stimuli (i.e., PFS-Food Available, PFS-Food Present, and PFS-Food Tasted) (40, 41). The Yale Food Addiction Scale (YFAS) assessed addiction-like feeding behavior (42) with a continuous score (number of symptoms, from 0 to 7) or diagnosis score. Furthermore, we used the Portuguese version of the Dutch Eating Behavior Questionnaire (DEBQ) (46, 47) to evaluate feeding behavior traits: emotional eating and restrained eating. Acceptance for food (fruits, vegetables, dairy, meat, fried, sauces, carbs, sweets) and alcohol was determined using the Food Action Rating Scale (FARS) (48).

### Statistical analysis

Categorical variables are represented as percentages, and continuous variables as mean and standard deviation (SD). A two-tailed *p*-value of 0.05 was selected as the significance level for all analyses. Statistical analyses were performed using SPSS^®^ version 26 (SPSS Inc., Chicago, IL, USA) and GraphPad Prism version 8.0 (GraphPad Software, La Jolla, CA, USA). Graphs were edited in Adobe Illustrator version CC 2019 (Adobe Inc., San Jose, CA, USA).

Our main analyses aimed to determine the effects of gustatory and psychometric variables on the likelihood of obesity. For unadjusted comparisons between groups, an independent-samples *t*-test or chi-squared test (χ^2^) was used, as appropriate. Cohen’s d (d) for the effect size of group differences was calculated as the mean difference between the two groups, divided by the pooled standard deviation. The primary analyses were multivariable logistic regressions while adjusting for confounders on taste perception or obesity, namely age, gender, and education (24, 36). Although standardized protocols across samples were used, the research center was added to the model to control any possible bias. Continuous independent variables of interest (EGM taste thresholds, taste acuity, intensity, and pleasantness ratings, PFS, YFAS, DEBQ, FARS scores, and age) were treated as z-scores allowing ORs to be compared between models. Nagelkerke’s *R*^2^ was assessed as a measure of effect size. We tested multicollinearity between independent variables in the model and inspected Cook’s distance to check the influence of outliers. We also examined the variable variance. Since there were no Food Addiction (diagnosis score) cases in the healthy group, we did not test this variable in multivariable logistic regressions.

We performed correction for multiple comparisons for the primary analyses (multivariable logistic regressions) within the pre-specified primary independent variables: mean sweet intensity and mean sweet pleasantness ratings; and within each group of variables: intensity ratings; pleasantness ratings; other taste assessment variables; hedonic hunger scores; food addiction; feeding behavior traits (DEBQ scores); Food Acceptance (FARS scores). The corrections for multiple comparisons were calculated according to Benjamini-Hochberg (49), assuming a false discovery rate (FDR) of 0.1. At an exploratory level, RM two-way ANOVAs with the Geisser Greenhouse correction were computed to compare the obesity and healthy groups for intensity and pleasantness ratings across tastants’ concentrations. Post-hoc Bonferroni multiple comparison tests were then computed for each ANOVA.

Principal Component Analysis (PCA) was used to explore associations between sweet taste perception and reward-related feeding behavior. After determining which variables were associated with obesity in multivariable logistic regressions adjusted for age, gender, and education, those variables were tested in a PCA with the joint sample, including all individuals with valid measures. With this analysis we aimed to determine how the variables associated with obesity would cluster in a multidimensional space and, specifically, if they reflected a single unitary component, or multiple separate components in the PCA space. The PCA’s suitability was tested by analyzing the overall Kaiser-Meyer-Olkin (KMO) measure and Bartlett’s test of sphericity. A Varimax orthogonal rotation was performed for interpretability.

## Results

### Reward-related gustatory and psychometric variables associated with obesity

Individuals in the obesity group (*N* = 246) were older (*P* < 0.0001, d = 1.0), had fewer years of formal education (*P* < 0.0001; d = −0.9) and had higher BMI (*P* < 0.0001; d = 4.5) when compared with the healthy group (*N* = 174; Table 1). In addition, the frequency of women was higher in the obesity group (*P* = 0.02), as was the frequency of T2DM (*P* < 0.0001), while the distribution of smokers was similar across groups (*P* = 0.1). Within the obesity group, pre-bariatric patients (*N* = 212) were older (*P* < 0.0001, d = 1.6), had a higher percentage of women (*P* = 0.01) and had a lower educational level (*P* = 0.02; d = −0.4) when compared with *“The biggest loser”* subgroup (*N* = 34; Supplementary Table 1). One pre-bariatric patient was under GLP-1 analogue treatment (Liraglutide, Victoza^®^). Sensitivity analyses showed that exclusion of this case does not impact our results (data not shown).

**TABLE 1:** Demographic characteristics of the study groups.

Characteristic	Obesity *N* = 246	Healthy *N* = 174	*P*-value^1^	Cohen’s *d*^2^
			
	Mean, SD or no. (%)			
Age, years	41.0 (11.2)	31.2 (9.2)	<0.0001	1.0
Women	201 (81.7%)	126 (72.4%)	0.02	N/A
Education, years	10.5 (4.1)	14.2 (3.8)	<0.0001	−0.9
T2DM	42 (17.1%)	0 (0%)	<0.0001	N/A
Smokers	58 (23.6%)	28 (16.1%)	0.1	N/A
BMI, Kg/m^2^	42.4 (5.4)	23.2 (2.7)	<0.0001	4.5

^1^Independent-samples *t*-test was performed for continuous variables and chi-square test for categorical variables.

^2^Cohen’s *d* was determined by calculating the mean difference between the obesity and healthy groups and dividing the result by the pooled standard deviations.

BMI, body mass index; T2DM, personal history of type 2 diabetes mellitus.

**FIGURE 1:**
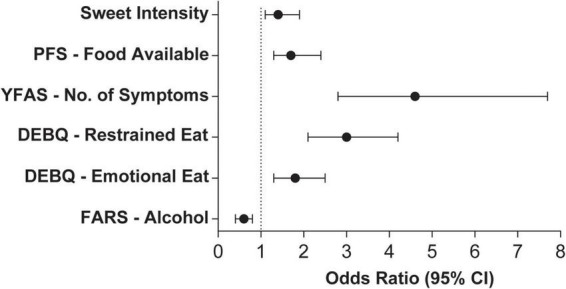
Odds ratio and 95% confidence intervals of gustatory and psychometric variables that were significantly associated with the obesity group. Models were adjusted for age, gender, education level, and research center. Independent variables were standardized to Z-scores for effect size comparison. *N* = 396. PFS, Power of Food Scale; YFAS, Yale Food Addiction Scale; DEBQ, Dutch Eating Behavior Questionnaire; FARS, Food Action Rating Scale.

Our primary hypothesis was that sweet taste perception is associated with obesity. We thus performed logistic regressions to assess the likelihood of belonging to the obesity group according to gustatory and psychometric variables when adjusting for age, gender, education, and research center. Of the gustatory variables tested, only sweet intensity ratings were associated with the obesity group (OR: 1.4, CI 95%: 1.1–1.9, *P* = 0.01), as shown in Figure 1 (please see Supplementary Table 2 for details). However, we did not find such association for hedonic ratings (i.e., sweet pleasantness), (OR: 1.2, CI 95%: 0.9–1.5, *P* = 0.2).

**FIGURE 2:**
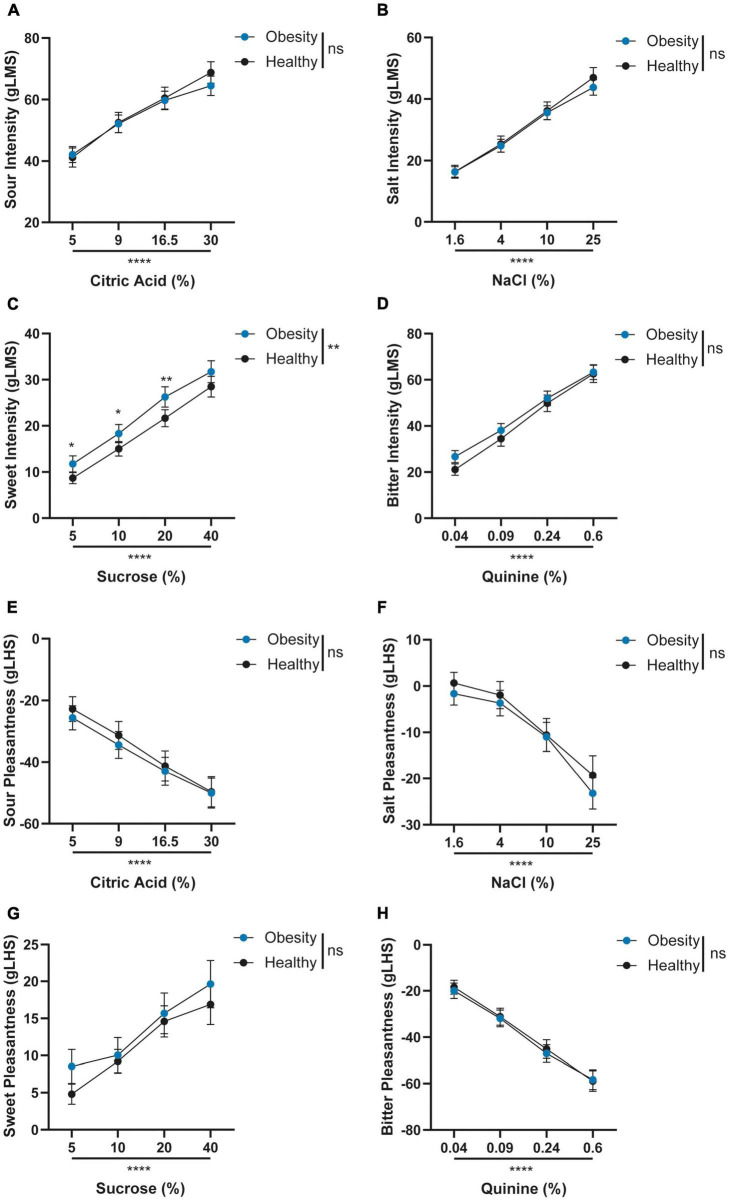
**(A–H)** Comparison of intensity and pleasantness ratings across tastants’ concentrations between obesity and healthy groups. Graphs represent means and 95% CI. Repeated measures two-way ANOVA with the Geisser Greenhouse correction and Bonferroni’s multiple comparison’s test were computed for each comparison. ns: *P* > 0.05; **P ≤ 0.01; and ****P ≤ 0.0001; Obesity, *N* = 230; and Healthy, *N* = 166. gLMS, general Labeled Magnitude Scale; gLHS, general Labeled Hedonic Scale.

Since we used the average sweet intensity ratings of the four sucrose concentrations in this analysis, we explored whether this result reflected differences in specific concentrations. Indeed, we found differences between the obesity and healthy groups for sweet intensity ratings but not for intensity or pleasantness ratings of the remaining tastants (Figures 2A–H), using RM two-way ANOVA. Furthermore, Bonferroni’s multiple comparison tests showed higher intensity ratings in the obesity group for 5, 10, and 20% sucrose (all *P* ≤ 0.05), but not for 40% sucrose (*P* = 0.2; Figure 2C).

Of the psychometric variables tested, those that were significantly associated with the likelihood of belonging to the obesity group were increased hedonic hunger (PFS–Food Available; OR: 1.7, CI 95%: 1.3–2.4, *P* = 0.001), increased number of addiction-like feeding behavior symptoms (YFAS–No. of symptoms; OR: 4.6, CI 95%: 2.8–7.6, *P* < 0.0001) and higher restrained eating (DEBQ–Restrained Eating; OR: 3.0, CI 95%: 2.1–4.2, *P* < 0.0001) as well emotional eating (DEBQ–Emotional Eating; OR: 1.8, CI 95%: 1.3–2.5, *P* = 0.001; Figure 1 and Supplementary Table 2). Conversely, alcohol acceptance was associated with a decreased likelihood of belonging to the obesity group (FARS-Alcohol; OR: 0.6, CI 95%: 0.4–0.8, *P* = 0.0002). Results of the previously described models remained significant after adjustment for multiple comparisons. Since the independent variables were standardized to Z-scores, the differences in effect size can be directly compared. Thus, the smallest differences were found in alcohol acceptance, sweet intensity perception, hedonic hunger, and emotional eating, followed by restrained eating and food addiction. Finally, in most logistic regression models, males had lower odds of belonging to the obesity group than females. Increasing age and lower education levels were also associated with an increased likelihood of belonging to the obesity group.

### Clusters of gustatory and reward-related feeding behavior variables

**FIGURE 3:**
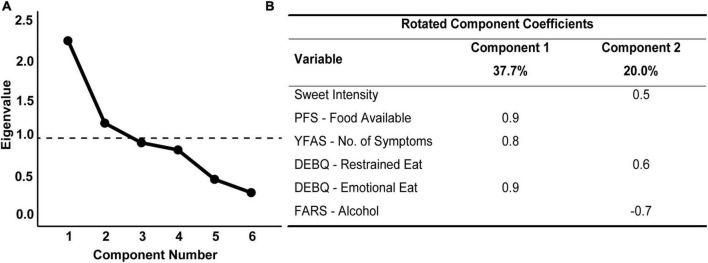
Scree plot **(A)** and rotated structure matrix **(B)** for principal component analysis of gustatory and psychometric variables associated with the obesity group, conducted in the joint sample. Method: Varimax with Kaizer normalization. Major loadings for each loading were kept. Loadings lower than 0.30 were suppressed. The principal component analysis was run with six variables that were previously associated with the obesity group, namely, sweet intensity, hedonic hunger (Power of Food Scale - Food Available), food addiction (Yale Food Addiction Scale - No. of Symptoms), restrained and emotional eating (Dutch Eating Behavior Questionnaire) and acceptance for alcohol (Food Action Rating Scale). *N* = 280.

After determining which variables were significantly associated with obesity, we aimed to test if sweet taste perception was related to measures of self-rated reward-related feeding behavior. We conducted a PCA with the variables associated with obesity (i.e., sweet intensity, hedonic hunger, food addiction, restrained and emotional eating, and acceptance of alcohol) in all individuals with valid measures (*N* = 280; Figure 3). Inspection of the correlation matrix showed that all variables had at least one correlation coefficient greater than 0.3 (data not shown). The overall KMO measure was 0.7, and Bartlett’s test of sphericity was statistically significant (*P* < 0.001), indicating that the data was factorizable. The PCA revealed two components with eigenvalues greater than one, and visual inspection of the scree plot (Figure 3A) indicated that the two components should be retained. Given that a two-component solution met the interpretability criterion, these components were retained (Figure 3B). This solution explained 57.7% of the total variance, with the first component explaining 37.7%. The latter included hedonic hunger (PFS–Food Available), food addiction (YFAS–No. of symptoms), as well as emotional eating (DEBQ–Emotional Eating). The second component explained 20% of the total variance and comprised sweet intensity perception, restrained eating (DEBQ–Restrained Eating), and acceptance of alcohol (FARS–Alcohol), as shown in Figure 3B.

## Discussion

The present study showed that sweet intensity perception is enhanced in obesity while addressing several limitations of previous research, namely the inclusion of a large sample size, a control group and adjustment for confounding variables. Furthermore, we found that sweet intensity perception varies independently of reward-related feeding behavior. Instead, it is inversely associated with alcohol acceptance and moderately associated with increased cognitive restraint of eating.

In agreement with our hypothesis, we found altered sweet taste perception in a sample of participants with obesity, specifically, increased sweet intensity ratings. This result was found across three of the four sucrose concentrations tested, showing consistent differences between obesity and healthy groups across sweet stimuli. However, no differences were found for sour, salt, or bitter tastants, suggesting specificity for sweet taste. Within the latter, we found differences in intensity but not hedonic ratings for sweet. A few studies corroborate that individuals with obesity perceive sweetness more intensely when compared with healthy individuals (29, 31). In a smaller sample, others showed that individuals with obesity, compared to non-obese subjects, had higher perceived intensity for the lower concentrations of sucrose (31) and lower thresholds for sucrose. However, this was also observed for salt and sour. In another study, adolescents with severe obesity showed higher perceived intensities at supra-threshold levels for sucrose and salt, along with lower recognition thresholds (higher sensitivity) to sucrose and sodium chloride relative to non-obese adolescents (29).

Contrary to our results, some studies found group differences in pleasantness rather than intensity (19). For example, Rodin et al. (28) showed that individuals with obesity or mild overweight rated higher concentrations of sweet as more pleasant relative to normal weight participants, using 9-point intensity and liking scales, in a sip-and-taste without swallowing method with glucose at 0.125–3 M. Others have reported results in the opposite direction, with individuals with obesity rating higher concentrations of sucrose as less pleasant than normal-weight controls using liking −4 to 4-point scales, also in a sip-and-taste without swallowing method, but with sucrose at 1.95–19.5% (w/v) (25). In this case, differences in intensity perception were also not found, using a magnitude estimation method (25). Similarly, group differences were not found in other studies using magnitude estimation in a sip-and-taste without swallowing method for intensity (21) or intensity and pleasantness estimation (20). Several factors may have determined variability in results relative to prior research, mainly methodological differences (19).

Enhanced sweet taste perception raises the possibility of increased central or peripheral sensitivity to sweet stimuli or learned associations with post-ingestive feedback of sugars in obesity. Wang et al. (50) compared individuals with severe obesity and non-obese controls using PET with 2-deoxy-2[^18^F] fluoro-D-glucose (FDG) to measure regional brain glucose metabolism, a proxy for neuronal activity. This study found increased activity in regions of the somatosensory cortex that process sensation to the mouth, lips, and tongue of individuals with obesity (50), supporting that individuals with obesity may have enhanced sensory sensitivity that contributes to their vulnerability to the reinforcing properties of food.

Since sweet intensity is a proxy for sugar concentration (24), enhanced sweet intensity can also reflect a learned preference for calories from simple carbohydrates. Indeed, there is pre-clinical evidence that preference for sugar is developed even in rodents that lack sweet taste receptors, showing a post-ingestive mechanism independent of taste (10). Post-ingestive reward has since been demonstrated through a direct infusion of sugar to the stomach (11), leading to food-seeking with associated increased activity of VTA dopamine neurons (11). Accordingly, healthy individuals develop small increases in preference for flavors paired with calories from carbohydrates (51), and metabolic response to carbohydrates is most significant when sweetness and caloric load are matched (52). Further evidence in healthy humans showed immediate and delayed dopamine release after a milkshake consumption in distinct brain areas, interpreted as dopamine release induced by orosensory and post-ingestive stimulation (53). These results indicate that post-ingestive signals may be primary for generating a reward response to sugars and supporting a potential role in for taste perception. Indeed it is known that sweet taste perception is influenced by peripheral factors, such as leptin (54) and glucagon-like peptide-1 (GLP-1) (55). However, to our knowledge, correlates of these hormones with measures of sweet taste in human studies have not been reported. The interplay between taste and post-ingestive signals is mainly unexplored in individuals with or without obesity. Further work on this subject may provide insight into the findings reported here.

The current study corroborates previous findings of increased self-reported sensitivity to food reward in obesity, measured by the PFS (40, 41, 43, 44, 56) and the YFAS (42, 45, 57, 58). Furthermore, our study provides novel information about the effect size of these differences between obesity and healthy groups. The effect size of hedonic hunger was very similar to our previous results of the association between the PFS–Food Available subscale and obesity status (44). Emotional eating followed this effect size, while food addiction symptoms were associated with an even higher likelihood of belonging to the obesity group. The fact that restrained and emotional, but not external, eating were associated with obesity is consistent with literature suggesting that only the latter reflects adaptive behavior to the environment (59). Psychometric measures of hedonic hunger, food addiction, and emotional eating were strongly correlated, in line with the previous literature (45, 60).

However, enhanced sweet taste perception was positively associated with restrained eating and inversely associated with alcohol acceptance, defining a distinct cluster of feeding-related features. In accordance to what is shown here, others had suggested that dietary restraint can vary independently from emotional eating (59). In addition, a sweet-alcohol relationship has been shown in a clinical trial of naltrexone for alcohol dependence (61). Indeed, in that study, patients with the sweet-liking phenotype and higher levels of craving for alcohol at baseline had fewer heavy drinking days when treated with naltrexone than with placebo (61). Thus it is possible that specific aspects of the neurocognitive vulnerability that characterizes obesity (62) may also contribute to sweet taste sensitivity, cognitive restraint of eating and alcohol consumption. This framework is particularly relevant when considering the increase in alcohol use disorders after bariatric surgery (63), for which changes in reward processing have been implicated (63). However, decreased alcohol acceptance in the obesity group should be carefully interpreted since it could merely corroborate findings of reduced alcohol use as the surgical date approached (64).

This study should be interpreted considering its limitations. Indeed, this study used a direct method for taste assessment comprising basic taste stimuli (i.e., sour, salt, sweet, and bitter), and standard scales to rate intensity and pleasantness perceptions. Thus, we must discuss our results primarily in the light of works using comparable methods. However, there is a large body of literature that instead of pure taste stimuli used foods or beverages and still did not find consistent associations between sensory hedonic pleasure and obesity (13, 14, 18). Importantly, this concept should be differentiated from other variables such as “preference” and “wanting”. The latter is typically described as the motivational component of reward (65, 66) and, in laboratory conditions, “wanting” for highly palatable foods is typically assessed using implicit measures such as reaction time (67), that are not necessarily accessible to conscious perception (65, 67, 68). In the present study, while we show a dissociation between sweet intensity and sweet pleasantness (i.e., a proxy for “liking”) on associations with obesity, we do not have “wanting” measures and thus, could not test if intensity perception is associated with this measure reflecting choice and action.

It should also be noted that the obesity group comprised pre-bariatric patients primarily. The inclusion of the “biggest loser” subgroup, which was, on average, younger, with more years of formal education, and had a higher percentage of males, may have attenuated potential effects associated to referral for surgery, but both groups had morbid obesity. We do not have data to assess, for example, if pre-bariatric patient counseling (medical, nutritional, and psychological support) influenced the consumption of sugars. However, this cannot be excluded since a study conducted on healthy subjects showed that sugar reduction resulted in increased perception of the intensity of sweet foods relative to controls after 2 months of diet (69).

Furthermore, specific aspects of diet, such as consumption of low-calorie sweeteners or type of diet (e.g., very low-calorie diet) that can impact taste perception, were not analyzed in this study, and may have impacted our results. Further studies in this field should include measures of dietary assessment (e.g., food diaries) to control for potential confounders. However, as described in a previous publication (33), within the pre-bariatric group, a subgroup remained in the waiting list for up to 18 months, while other subgroup was tested just prior to bariatric surgery. These sub-groups did not differ in sweet intensity ratings (33). Considering that patients are more likely to be under energy restriction in the weeks before bariatric surgery, this suggests that energy restriction was not a major contributor to the response to sweet taste among patients with obesity.

Finally, alcohol abuse was an exclusion criterion in this study, which does not allow generalization of our results about decreased alcohol acceptance to obesity.

## Conclusion

Our findings support enhanced sweet taste perception in obesity while addressing critical methodological limitations of previous studies. Our study also corroborates increased reward-related feeding behavior in obesity. However, enhanced sweet intensity perception was not associated with psychological measures of reward-related feeding behavior but with elevated restrained eating and reduced alcohol acceptance. Further study of sweet taste perception and its correlates in obesity is needed to clarify the role of sweet taste in human obesity.

## Data availability statement

The raw data supporting the conclusions of this article will be made available by the authors, under reasonable request.

## Ethics statement

The studies involving human participants were reviewed and approved by Ethics Committees in the several institutions involved in the study. Approved 24/09/2012, Comissão de Ética - Área da Saúde Humana e Bem-Estar (Universidade de Évora. Largo dos Colegiais 2, 7000-645 Évora, Portugal; + 351 (0)266 740 800; comissao.etica@uevora.pt), ref: 12031 2. Approved 22/07/2013, Comissão de Ética da Fundação Champalimaud (Fundação Champalimaud. Avenida Brasília 1400-038 Lisboa, Portugal; + 351 (0)210 480 200; info@fundacaochampalimaud.pt), ref: N/A 3. Approved 05/12/2013, Comissão de Ética para a Saúde do Centro Hospitalar de São João E.P.E. (Alameda Professor Hernâni Monteiro 4200-319 Porto, Portugal; + 351 (0)225 512 100; geral@hsjao.min-saude.pt), ref: CES254-13 4. Approved 06/08/2014, Conselho de Administração do Centro Hospitalar de Setúbal E.P.E. (Rua camilo castelo Branco 2910-446, Setúbal, Portugal; + 351 (0)265 549 000; geral@chs.min-saude.pt), ref: 280/C.A. The patients/participants provided their written informed consent to participate in this study.

## Author contributions

AJO-M concept and designed and obtained funding. GR and AJO-M drafted the manuscript. All authors performed acquisition, analysis, or interpretation of data and critical revision of the manuscript for intellectual content.
