# Supplementary material for: Enhanced sweet taste perception in obesity: Joint analysis of gustatory data from multiple studies
Source: Front Nutr. 2022 Dec 20;9:1028261. doi: 10.3389/fnut.2022.1028261 (PMC9807659; doi:10.3389/fnut.2022.1028261)
Supplement: Supplementary file 1 [file Presentation_1.pdf]

Supplementary Information

Enhanced sweet taste perception in obesity: joint analysis of gustatory data from multiple studies.

Gabriela Ribeiro, Sandra Torres, Ana B. Fernandes, Marta Camacho, Teresa L. Branco, Sandra S. Martins, Armando Raimundo, Albino J. Oliveira-Maia, and Food Reward in Bariatric Surgery Portuguese Study Group.

1. **Supplementary Methods**
   1.1. Protocol for preparation of the in-house taste strips test.
   1.2. Reliability assessment of the in-house taste strip test.
      1.2.1. Methods
      1.2.2. Results
      1.2.3. Discussion

2. **Supplementary Tables**
   Supplementary Table 1. Demographic, gustatory, and feeding behavior variables between subgroups of the obesity group.
   Supplementary Table 2. Gustatory and feeding behavior variables that explained the likelihood of obesity vs. healthy group belonging.

3. **Supplementary References**

1. Supplementary Methods

1.1. Protocol for preparation of the in-house taste strips test.

Four basic tastants (sour, salt, sweet and bitter) were prepared in deionized water (Milli-Q, Millipore) at 4 different concentrations Sour: 0.05, 0.09, 0.0165 and 0.3g/ml of citric acid; Salt: 0.016, 0.04, 0.1 and 0.25g/ml of sodium chloride; Sweet: 0.05, 0.1, 0.2 and 0.4 g/ml of sucrose; Bitter: 0.0004, 0.0009, 0.0024 and 0.006 g/ml of quinine hydrochloride. Citric acid, sodium chloride, sucrose, and quinine hydrochloride were purchased from Sigma Aldrich. Wide strips of filter paper (GE Healthcare Life Sciences Whatman™, diameter 320mm) were soaked in the previously prepared solutions and dried at room temperature. After drying, filter paper strips were cut in a rectangle shape (average of 3.0 cm x 2.0 cm) and stored in separated sealed plastic bags for each tasting concentration. Solutions and taste strips were prepared freshly in regular intervals of 3 months.

1.2. Reliability assessment of the in-house taste strip test

1.2.1. Methods

We tested the agreement of our in-house taste strips test with a similar commercially available test (Burghart Messtechnik GmbH, Wedel, Germany). We aimed to determine if minor methodological differences would impact results. The in-house and the commercially available tests comprise the same tastants at the same concentrations. However, the area of the taste strip of the in-house test is larger, and the paper filter is thinner and more malleable. We tested the temporal reliability of the in-house method to ensure it would be suitable for longitudinal studies. We applied both tests to a group of

26 healthy volunteers. Equivalent protocols were used for both tests, as described above, including the gLMS[1] and gLHS[2]. For temporal reliability estimation of the in-house test, volunteers were evaluated approximately one month after baseline in the same conditions. We chose this interval because it is sufficient to minimize recall but not too long for the occurrence of significant events across most participants.

1.2.2. Statistical Analysis

We performed repeated measures (RM) two-way analysis of variance (ANOVA) with the Geisser-Greenhouse correction to compare the two tests regarding intensity and pleasantness ratings across tastants' concentrations with Bonferroni multiple comparison tests. The intraclass correlation coefficient (ICC) estimates, and their 95% confidence intervals (CI) were calculated based on an average-measures, absolute-agreement, 2-way mixed-effects model for temporal reliability assessment. Regarding agreement analysis, we performed the gold standard Bland-Altman plot and analysis to compare the two tests (i.e., in-house, and commercially available) regarding intensity and pleasantness ratings.

1.2.3. Results

The in-house test resulted in higher acuity and higher mean intensity ratings for all tastants (**Methodological Figure 1 A-E**; all $P \leq 0.001$). And lower mean pleasantness ratings for sour and bitter and higher mean pleasantness ratings for sweet taste (**Methodological Figure 1 F-I**). We then compared the two tests regarding intensity and pleasantness ratings for each of the concentrations tested. We found that the in-house test resulted in higher intensity ratings for most tastant concentrations, with very few exceptions (e.g., 5% sucrose; **Methodological Figure 2 A-D**). Regarding pleasantness, the in-house test resulted in lower pleasantness ratings across most concentrations of non-

appetitive tastants (i.e., sour and bitter). In addition, there were higher pleasantness ratings for several concentrations of sweet. However, we did not find differences between the two methods regarding salt pleasantness ratings for any of the concentrations (**Methodological Figure 2 E-H).** Finally, the in-house and commercially available agreement was tested using the gold standard Bland-Altman plot and analysis. We found an overall poor agreement in intensity and pleasantness ratings (**Methodological Figure 3**) between the two tests, with the average mean differences or 'bias' (represented by the weighted line) being far from zero. Generally, points did not form uniform bands across the graphs but rather trends suggesting proportional error. Salt pleasantness ratings were the only parameter that showed an average bias close to zero (**Methodological Figure 3 F**). The in-house test was also applied a second time in the same individuals (N=26), approximately one month after the baseline assessment [4.8 (0.4) weeks]. Acuity was the only parameter that showed a poor agreement between estimates (ICC=0.1, P=0.4). ICC values for intensity and pleasantness ratings ranged from 0.5 to 0.8 (all P≤0.05), corresponding to moderate to good agreement between assessments (**Methodological Table 1**).

1.2.4. Discussion

We showed that two similar taste tests (in-house vs commercially available), applied under the same conditions (i.e., the same application method, stimuli, concentrations, and reporting scales), resulted in similar but not interchangeable results. Overall, the two tests seem to measure the same outcomes. However, the in-house test resulted in higher intensity ratings for appetitive tastants and lower pleasantness ratings for non-appetitive tastants (except for salt). Since we used the same concentrations, the larger area of the taste strip or the paper filter weight and thickness may have contributed to these

differences. Furthermore, we showed that the in-house test has appropriate temporal reliability and, thus, is suitable for longitudinal studies. Nevertheless, it is also clear that even slight methodological differences can impact the observed results.

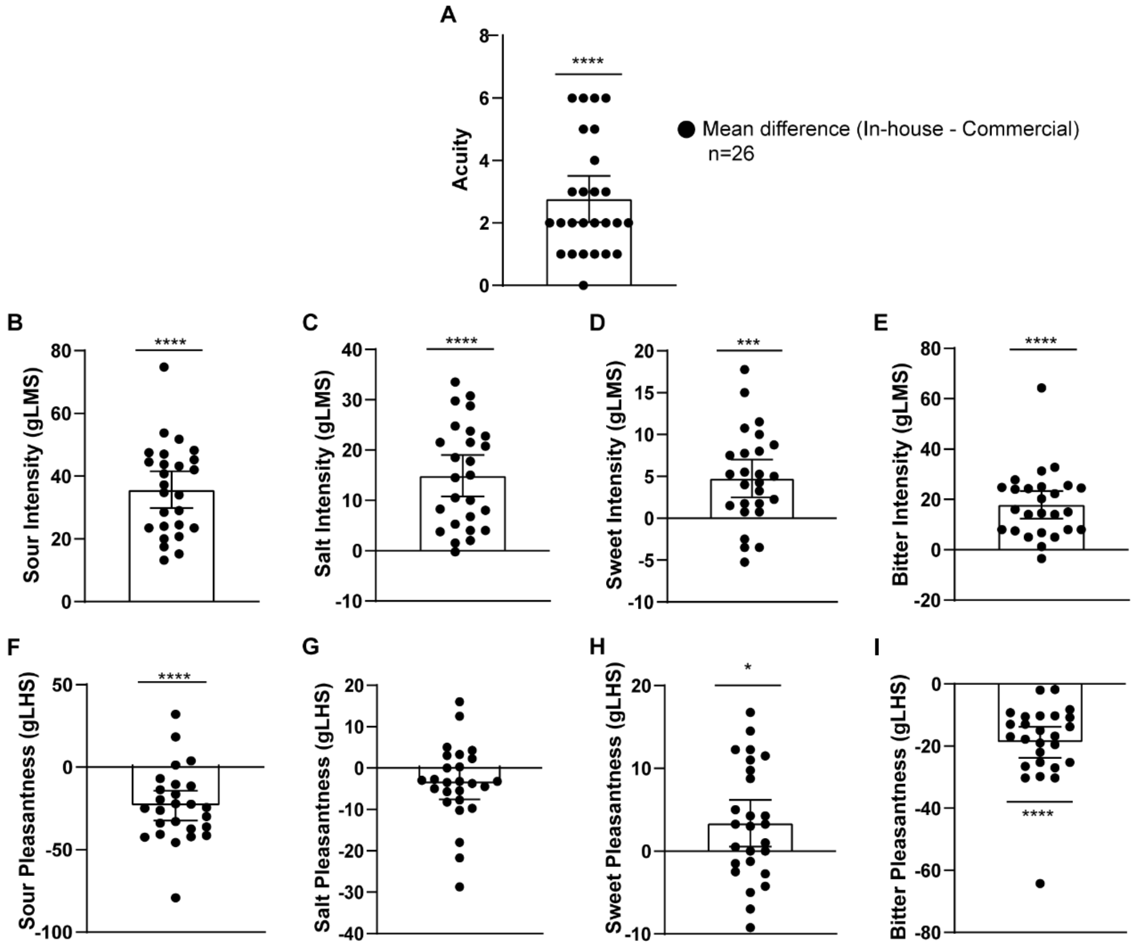

Methodological Figure 1. **Comparison between in-house and a commercially available taste strips test across mean intensity and pleasantness ratings.**

Notes: Graphs represent mean differences and 95 % Confidence Intervals. P values are the result of one-sample t-tests. *P ≤ 0.05; ***P ≤ 0.001; ****P ≤ 0.0001. N=26.

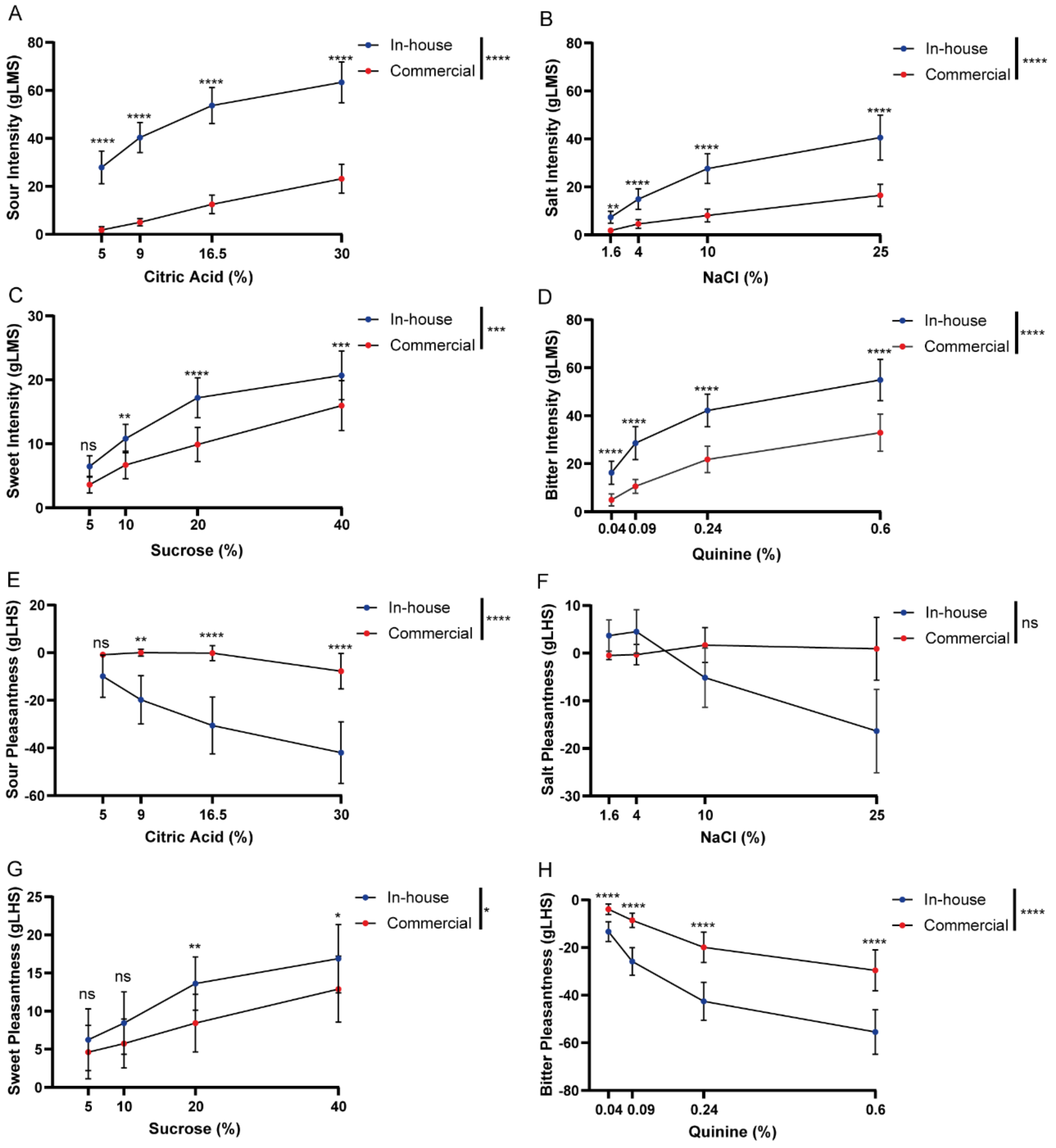

Methodological Figure 2. Comparison between in-house and a commercially available taste strips test across intensity and pleasantness ratings for all concentrations.

Notes: Graphs represent means and 95% Confidence Intervals. Repeated Measures two-way ANOVA with the Geisser Greenhouse correction and Bonferroni's multiple comparison's test were computed for each comparison. *P ≤ 0.05; **P ≤ 0.01; ***P ≤ 0.001; ****P ≤ 0.0001; N=26.

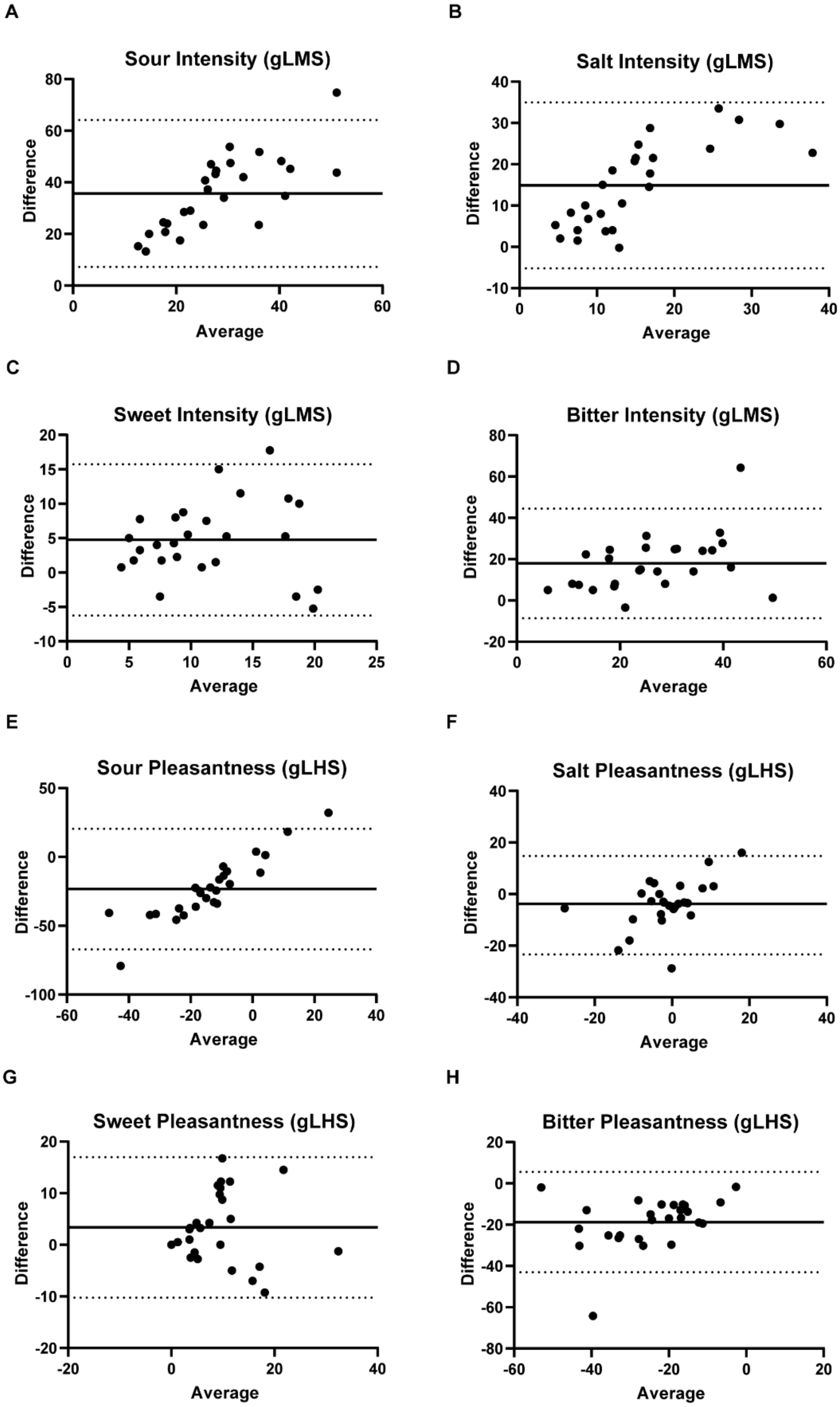

Methodological Figure 3. Bland-Altman analysis for agreement testing between in-house and a commercially available taste strips test.

Notes: Graphs represent the mean difference between in house and commercially available taste strip tests against the average values of the two methods in the same individuals. The weighted line represents the average of the mean differences while the dotted lines represent the 95% lower and upper limits of agreement. N=26.

Methodological Table 1. Temporal reliability of the 'in-house' taste strips test.

Variable	Assessment I	Assessment II	ICC	95% CI		*P-Value*
	Mean, SD; N=26			**Lower Bound**	**Upper Bound**	
Acuity	15.1 (1.5)	15.6 (0.6)	0.1	-0.98	0.6	0.4
Sour ratings, *mm*						
Intensity	34.2 (19.2)	46.3 (16.8)	0.5	0.001	0.8	0.01
Pleasantness	-28.5 (27.2)	-25.5 (26.0)	0.8	0.5	0.9	0.0004
Salt ratings, *mm*						
Intensity	27.0 (10.7)	22.6 (12.9)	0.7	0.4	0.9	0.001
Pleasantness	-4.7 (11.7)	-3.3 (11.9)	0.7	0.3	0.9	0.003
Sweet ratings, *mm*						
Intensity	16.5 (7.5)	13.8 (5.9)	0.7	0.3	0.8	0.002
Pleasantness	10.7 (9.2)	11.3 (7.7)	0.7	0.3	0.9	0.002
Bitter ratings, *mm*						
Intensity	40.2 (15.4)	35.5 (15.5)	0.7	0.3	0.9	0.002
Pleasantness	-34.9 (16.6)	-34.3 (15.8)	0.7	0.2	0.9	0.05

The interval between the first and second assessments was 4.8 (0.4) weeks.
The Intraclass Correlation Coefficient (ICC) estimates and their 95% confidence intervals were calculated based on an average-measures, absolute-agreement. 2-way mixed-effects model.
Based on the 95% confidence interval of the ICC estimate, the next cut-off values were established: less than 0.5 (poor), between 0.5 and 0.75 (moderate), between 0.75 and 0.9 (good), and greater than 0.90 (excellent).

Supplementary Table 1. Demographic, gustatory, and feeding behavior variables between subgroups of the obesity group.

	Obesity group			
Variable	Pre-bariatric N=212	The biggest Loser N=34	*P Value*[2]	Cohen's d[3]
	Mean, SD or No. (%)[1]			
Age, years	43.2 (10.2)	27.4 (7.0)	<0.0001	1.6
Women	180 (84.9)	21 (61.8)	0.01	N/A
Education, *years*	10.2 (4.1)	11.9 (3.4)	0.02	-0.4
T2DM	42 (19.8)	0 (0)	0.004	N/A
Smokers	46 (21.7)	12 (35.3)	0.1	N/A
BMI, Kg/m^2	42.7 (5.3)	41.0 (6.0)	0.1	0.3
Taste thresholds, dB	18.5 (14.6)	10.2 (11.7)	0.002[4]	0.6
Acuity	12.5 (2.8)	13.5 (2.6)	0.1	-0.4
Sour ratings, *mm*				
Intensity	54.0 (18.9)	58.3 (22.2)	0.2	-0.2
Pleasantness	-38.9 (29.6)	-34.9 (35.2)	0.5	-0.1
Salt ratings, *mm*				
Intensity	29.8 (13.4)	31.7 (14.1)	0.5	-0.1
Pleasantness	-10.2 (17.5)	-7.9 (21.0)	0.5	-0.1
Sweet ratings, *mm*				
Intensity	21.7 (13.1)	23.7 (15.4)	0.4	-0.2
Pleasantness	12.6 (15.1)	18.7 (21.1)	0.04[4]	-0.4
Bitter ratings, *mm*				
Intensity	44.1 (18.5)	50.6 (21.0)	0.1	-0.3
Pleasantness	-38.6 (22.7)	-43.0 (22.9)	0.3	0.2

[1]Results are presented as mean and standard deviation (SD) or number and percentage (%).
[2]Independent sample t-tests were performed for continuous variables and $\chi 2$ tests for categorical variables compared to obesity and healthy groups.
[3]Cohen's d was determined by calculating the mean difference between pre-bariatric and 'The biggest looser' groups and dividing the result by the pooled standard deviations.
[4]The differences don't subsist after adjustment for age in binary logistic models.
Abbreviations: T2DM: a personal history of Type 2 Diabetes *Mellitus*

Supplementary Table 2. Gustatory and feeding behavior variables that explained the likelihood of obesity vs. healthy group belonging.

Variable	Obesity	Healthy	*P* Value[2]	Cohen's d[3]	Adjusted OR[4], 95% CI	*P*-Value
	Mean, SD or No. (%)[1]				N=396[5]	
Taste thresholds, *dB*	17.2 (14.4)	9.8 (13.1)	<0.0001	0.5	1.2 (0.9 - 1.6)	0.1
Acuity	12.6 (2.8)	13.7 (2.4)	<0.0001	-0.4	0.8 (0.6 - 1.1)	0.1
Sour ratings, *mm*						
Intensity	54.6 (19.4)	53.2 (21.2)	0.5	0.1	1.2 (0.9 - 1.5)	0.2
Pleasantness	-38.3 (30.5)	-36.3 (27.0)	0.5	-0.1	0.9 (0.7 - 1.2)	0.5
Salt ratings, *mm*						
Intensity	30.1 (13.5)	30.5 (15.6)	0.8	0.0	1.0 (0.8 - 1.2)	0.7
Pleasantness	-9.9 (18.0)	-7.2 (19.0)	0.1	-0.2	0.9 (0.7 - 1.1)	0.2
Sweet ratings, *mm*						
Intensity	22.0 (13.5)	18.5 (9.5)	0.004	0.3	1.4 (1.1 - 1.9)	0.01
Pleasantness	13.5 (16.2)	11.4 (10.4)	0.1	0.2	1.2 (0.9 - 1.5)	0.2
Bitter ratings, *mm*						
Intensity	45.0 (19.0)	42.0 (18.9)	0.1	0.2	1.3 (0.99 - 1.6)	0.1
Pleasantness	-39.3 (22.7)	-38.4 (21.1)	0.7	0.0	0.9 (0.7 - 1.2)	0.5

Reward-related feeding behavior						
PFS - Aggregate score	2.3 (0.8)	2.2 (0.7)	0.3	0.1	1.3 (0.97 - 1.8)	0.1
PFS - Food Available	2.1 (0.9)	1.8 (0.6)	0.003	0.4	1.7 (1.3 - 2.4)	0.001
PFS - Food Present	2.7 (1.1)	2.7 (1.0)	0.8	0.0	1.0 (0.8 - 1.4)	0.9
PFS - Food Tasted	2.4 (0.9)	2.6 (0.9)	0.04	-0.2	0.9 (0.7 - 1.2)	0.4
YFAS - Diagnosis	48 (23.5%)	0 (0%)	<0.0001	N/A	N/A[6]	
YFAS - No. of Symptoms	2.7 (1.8)	1.4 (1.0)	<0.0001	0.9	4.6 (2.8 - 7.6)	<0.0001
Feeding behavior traits						
DEBQ - External Eat	2.5 (0.7)	2.7 (0.5)	<0.05	-0.2	1.1 (0.8 - 1.4)	0.8
DEBQ - Restrained Eat	3.0 (0.7)	2.3 (0.8)	<0.0001	1.0	3.0 (2.1 - 4.2)	<0.0001
DEBQ - Emotional Eat	2.2 (0.9)	1.9 (0.7)	0.003	0.3	1.8 (1.3 - 2.5)	0.001
Food Acceptance						
FARS - Aggregate score	410.0 (54.2)	397.1 (46.0)	0.04	0.3	1.0 (0.7 - 1.4)	1.0
FARS - Fruit	71.1 (11.5)	67.5 (11.0)	0.01	0.3	1.2 (0.9 - 1.6)	0.1
FARS - Vegetables	102.4 (18.9)	93.8 (17.5)	0.0001	0.5	1.0 (0.7 - 1.4)	0.9
FARS - Dairy	21.4 (4.3)	20.0 (4.0)	0.004	0.3	1.1 (0.9 - 1.5)	0.4
FARS - Meat	38.4 (6.8)	36.9 (7.2)	0.1	0.2	1.1 (0.8 - 1.5)	0.5
FARS - Fried	25.5 (7.8)	25.9 (5.4)	0.6	-0.1	0.9 (0.6 - 1.2)	0.3

FARS - Sauce	20.0 (6.2)	20.1 (5.1)	0.9	0.0	1.0 (0.8 - 1.4)	0.8
FARS - Carbs	62.9 (10.6)	60.7 (9.1)	0.1	0.2	1.2 (0.9 - 1.7)	0.1
FARS - Sweets	34.7 (9.1)	35.5 (7.5)	0.4	-0.1	0.9 (0.7 - 1.2)	0.6
FARS - Alcohol	8.8 (5.5)	11.9 (5.8)	<0.0001	-0.6	0.6 (0.4 - 0.8)	0.0002

[1]Results are presented as mean and standard deviation (SD) or number and percentage (%).

[2]Independent sample t-tests were performed for continuous variables and $\chi 2$ tests for categorical variables compared to obesity and healthy groups.

[3]Cohen's d was determined by calculating the mean difference between the obesity and healthy groups and dividing the result by the pooled standard deviations.

[4]Odds Ratio was adjusted for age, gender, education, and research center. Independent continuous variables in the binary logistic regression were standardized to z-scores.

[5]The sample size (N) represents the maximal number of participants with valid gustatory or psychometric measures.

[6]The diagnosis version Yale Food Addiction Scale results were not presented since there are no diagnosis cases in the non-clinical sample.

The Nagelkerke's R^2 of the binary logistic regression models ranged from 0.3 to 0.6.

Abbreviations: DEBQ - Dutch Eating Behavior Questionnaire; FARS - Food Action Rating Scale; PFS - Power of Food Scale; YFAS - Yale Food Addiction Scale.

Supplementary References

1. Green BG, Dalton P, Cowart B, Shaffer G, Rankin K, Higgins J. Evaluating the ‘Labeled Magnitude Scale’for measuring sensations of taste and smell. *Chem Senses*. 1996;21(3):323-334.

2. Lim J, Wood A, Green BG. Derivation and evaluation of a labeled hedonic scale. *Chem Senses*. 2009;34(9):739-751.
