# Supplementary material for: Enhanced sweet taste perception in obesity: Joint analysis of gustatory data from multiple studies
Source: Front Nutr. 2022 Dec 20;9:1028261. doi: 10.3389/fnut.2022.1028261 (PMC9807659; doi:10.3389/fnut.2022.1028261)
Supplement: Supplementary file 2 [file Data_Sheet_2.pdf]

Enhanced sweet taste perception in obesity: joint analysis of gustatory data from multiple studies. Ribeiro G., et al.

Supplementary Material

Appendix 1 - Food Reward in Bariatric Surgery Portuguese Study Group.

Principal investigator, responsible for the design, analysis and report of the study:

Prof. Albino J. Oliveira-Maia, psychiatrist and director of the Neuropsychiatry Unit at the Champalimaud Research and Clinical Centre, professor of Psychiatry and Neuroscience at NOVA Medical School, Faculdade de Ciências Médicas, NMS, FCM, Universidade NOVA de Lisboa, Lisbon, Portugal.

Co-investigators:

Professor Gabriela Ribeiro, clinical nutritionist and Invited Assistant Professor of Nutrition and Metabolism at NOVA Medical School, Faculdade de Ciências Médicas, NMS, FCM, Universidade NOVA de Lisboa, Lisbon, Portugal; Ms. Marta Camacho, neuropsychologist and research assistant at the Champalimaud Research and Clinical Centre, Lisbon, Portugal. Prof. Ana Fernandes, postdoctoral associate at Champalimaud Research and Clinical Centre and professor of Neuroscience at NOVA Medical School, Faculdade de Ciências Médicas, NMS, FCM, Universidade NOVA de Lisboa, Lisbon, Portugal; Gonçalo Cotovio, PhD Fellow at the Champalimaud Research and Clinical Centre and at the Biomedicine PhD Program, NOVA Medical School, Faculdade de Ciências Médicas, NMS, FCM, Universidade NOVA de Lisboa, Lisbon, Portugal. Psychiatry resident at the Department of Psychiatry, Centro Hospitalar Lisboa Ocidental E.P.E., Lisbon, Portugal; Professor Sandra Torres, psychologist and professor of Psychology at Faculdade de Psicologia e de Ciências da Educação and Centro de Psicologia, Universidade do Porto, Porto, Portugal.

Participating clinicians (alphabetical order):

André Ferreira, clinical psychologist at the Obesity Surgical Treatment Center, Hospital do Espírito Santo, E.P.E., Évora, Portugal; António Roma-Torres, retired psychiatrist and director of the Department of Psychiatry, Centro Hospitalar Universitário de S. João E.P.E., Porto, Portugal, and professor of Psychiatry at Faculdade de Medicina, Universidade do Porto, Porto, Portugal; Clotilde Limbert, Endocrinologist at the Department of Endocrinology, Hospital Egas Moniz. Centro Hospitalar de Lisboa Ocidental E.P.E., Lisboa. Portugal; Cristina Pontes, psychologist at the Department of Psychiatry, Centro Hospitalar Universitário de S. João, E.P.E., Porto, Portugal; Isabel Brandão, psychiatrist at the Department of Psychiatry, Centro Hospitalar Universitário de S. João, E.P.E., and professor of Psychiatry at Faculdade de Medicina, Universidade do Porto, Porto, Portugal; João Sequeira Duarte, Endocrinologist at the Department of Endocrinology, Hospital Egas Moniz. Centro Hospitalar de Lisboa Ocidental E.P.E., Lisboa. Portugal; Lia de Jesus, dietician at the Obesity Surgical Treatment Unit, Hospital de S. Bernardo, E.P.E., Setúbal, Portugal; Manuela Espírito Santo, clinical psychologist at the Obesity Surgical Treatment Unit, Hospital de S. Bernardo E.P.E., Setúbal, Portugal; Margarida Pegacho, nurse at the Obesity Surgical Treatment Center, Hospital do Espírito Santo, E.P.E., Évora, Portugal; Maria Oliveira, Endocrinologist at the Department of Endocrinology, Hospital Egas Moniz. Centro Hospitalar de Lisboa Ocidental E.P.E., Lisboa. Portugal; Nuno Nunes, dietician at the Obesity Surgical Treatment Unit, Hospital de S. Bernardo E.P.E., Setúbal, Portugal;

Participating surgeons (alphabetical order):

Ana André, general surgeon at the Obesity Surgical Treatment Unit, Hospital de S. Bernardo E.P.E., Setúbal, Portugal; Carlos Trindade, general surgeon at the Obesity Surgical Treatment Unit, Hospital de S. Bernardo E.P.E., Setúbal, Portugal; Eduardo Lima da Costa, general surgeon, surgery department, Centro Hospitalar Universitário de S. João E.P.E., Porto, Portugal; André Pinho, general surgeon, surgery department, Centro Hospitalar Universitário de S. João E.P.E., Porto, Portugal; Hugo Sousa, general surgeon, surgery department, Centro Hospitalar Universitário de S. João E.P.E., Porto, Portugal; John Preto, general surgeon, surgery department, Centro Hospitalar Universitário de S. João E.P.E., Porto, Portugal; José Adelino Barbosa, general surgeon, surgery department, Centro Hospitalar Universitário de S. João E.P.E., Porto, Portugal; Luís Cortez, general surgeon of the Obesity Surgical Treatment Unit, Director of the Surgery Department at the Hospital de S. Bernardo E.P.E., Setúbal, Portugal; Manuel Carvalho, general surgeon at the Obesity Surgical Treatment Center Hospital do Espírito Santo, E.P.E., Évora, Portugal; Silvestre Carneiro, general surgeon, surgery department, Centro Hospitalar Universitário de S. João E.P.E., Porto, Portugal;
